# The Malnutrition Universal Screening Tool (MUST) and a nutrition education program for high risk cancer patients: strategies to improve dietary intake in cancer patients

**DOI:** 10.7603/s40681-015-0017-6

**Published:** 2015-08-08

**Authors:** Pei-Chun Chao, Hui-Ju Chuang, Li-Yen Tsao, Pei-Ying Chen, Chia-Fen Hsu, Hsing-Chun Lin, Chiu-Yueh Chang, Cheau-Feng Lin

**Affiliations:** 1 School of Health Diet and Industry Management, Chung Shan Medical University, 402 Taichung, Taiwan; 2 Department of Nutrition, Chung Shan Medical University Hospital, 402 Taichung, Taiwan; 3 Department of Parenteral Nutrition, Chung Shan Medical University Hospital, 402 Taichung, Taiwan

**Keywords:** Oncology, Malnutrition, Nutrition education

## Abstract

Four hundred and forty-four high-risk oncology patients with malnutrition participated in this study aimed at assessing the effectiveness of nutrition education on improving an oncology patient’s dietary intake. We used a nutritional risk screening to select oncology patients in need of nutritional care. Team Nutrition provides technical assistance for foodservice, nutrition education for patients and their caregivers, and support for healthy eating and physical activity to improve their diets and their lives. The average contribution of protein and total energy of each patient increased after imparting the nutritional education to them. Thus, nutritional education is an effective measure to bring about a favorable and significant change in oncology patients’ nutrient intake.

## 1. Introduction

The death certification system in Taiwan has been computerized since 1971. Cancer has been the number one cause of death in Taiwan for decades [1]. Cancer-associated malnutrition has many consequences, including increased risk of infection, reduced wound healing, reduced muscle function, and poor skin turgor resulting in skin breakdown [2]. Nutritional support is recommended for malnourished people who are unable to maintain body weight by appetite and food intake often in the decline of a disease. Consequently, tailored strategies to identify patients at nutritional risk are essential to implement nutritional support effectively and to reduce cancer morbidity.

Routine screening for malnutrition should be implemented for people in at-risk groups. The risk of malnutrition and its severity in oncology patients are affected by the tumor type, stage of disease, and the antineoplastic therapy applied [3]. There are many valuable tools that have been developed, validated, and are currently widely used for the detection of malnutrition in clinical practice, including Subjective Global Assessment [4], MiniNutritional Assessment (MNA) [5] and its short form (SF-MNA) [6], Nutrition Risk Screening [7] and ‘Malnutrition Universal Screening Tool’ (MUST) [8].

This investigation selected a validated tool that was an easy and simple to screen patients at nutritional risk in oncology, MUST. MUST is a screening tool that has shown its strength for application with adult patients across all healthcare settings including oncology [9]. MUST is a five-step screening tool to identify patients who are malnourished and at risk of malnutrition (or undernutrition). Some strategies can be adopted to improve the nutritional status of these patients. These strategies include patient nutrition education programs, and the use of oral nutritional supplements, which can significantly impact nutritional status [10].

The standard treatment of undernutrition aims to achieve optimal protein and energy intake, according to a patient’s requirements, in order to reduce the effects of catabolism and minimize the loss of the body’s protein mass [11]. The objective of this study is to evaluate if there is a benefit to nutrition education and oral nutritional supplementation on the nutritional status of patients with cancer that are at a high risk of malnutrition.

## 2. Methods

### 2.1. Subjects

This is a chart review retrospective cross-sectional observation study that was approved by the Institutional Review Board with patients and their families signing waivers of informed consent. Patients were routinely screened with MUST. All cancer patients (n = 444) admitted to the hospital from January, 2011 to December, 2012 who were screened as undernourished (MUST score ≧ 2) at hospital admission were retrospectively included in this study. Patients below the age of 18 years or those who did not complete the nutrition education follow-up were excluded.

This study was conducted at the Chung Shan Medical University Hospital’s (Taichung, Taiwan) cancer care ward. The study was conducted according to the guidelines laid down in the Declaration of Helsinki, and all procedures involving human subjects and patient recruitment were approved by Institutional Review Board of the Chung Shan Medical University Hospital Review Board (CSMUH IRB No: CS11124).

### 2.2. Malnutrition screening tools

**Fig. 1:**
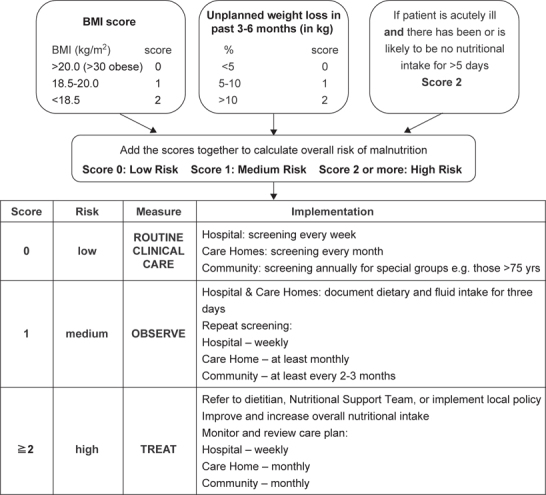
- Malnutrition Universal Screening Tool (MUST).

The purpose of the MUST system is to detect patients who are at risk for malnutrition or who are malnourished on the basis of knowledge about the association between impaired nutritional status, body composition, and physical function (Figure 1, see www.bapen.org.uk for a free download of tool and an explanatory booklet) [12].

Three independent criteria are used by MUST to determine the overall risk for malnutrition: current weight status using BMI, unintentional weight loss, and acute disease effect that has induced a phase of nil per os for > 5 days. Each parameter can be rated as 0, 1, or 2. Overall risk for malnutrition is established as low (score = 0), medium (score = 1), or high (score > 2). Each of these three criteria can independently predict a clinical outcome, varying by the clinical circumstance, but together the three criteria are better predictors than each by itself [9].

### 2.3. Study design

**Fig. 2:**
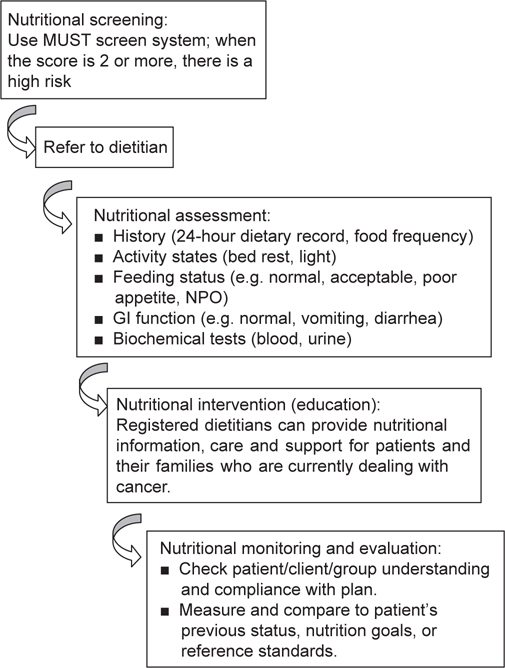
- Screening identifies cancer patients at high risk of malnutrition, who should then be referred to registered dietitians for optimal nutritional education.

The number of MUST scores undertaken by an experienced clinical nurse on patients within 24 h of admission. Data gathered included the numbers of patients with accurately measured height, weight, body mass index, weight loss, and acute disease effect scores. When a MUST score ≧ 2 was calculated, the patient was referred to a dietitian. Step 1 was to give a nutrition assessment and to ascertain from the patient themself, their caregiver(s), and food charts the patient’s past and present appetite and dietary intake, food likes/dislikes, factors affecting nutritional intake, weight history (current, previous, or any weight loss). Then, to go a step further, to formulate a dietetic care plan that involved food selection and meal planning patterns (using oral diet, extra snacks, and possible prescribed supplements). This information was collected before the intervention (baseline) and after the intervention (follow-up) to assess the effectiveness of nutrition education on improving the patient’s dietary intake and nutritional knowledge (Figure 2).

### 2.4. Data collection and processing

Meal patterns were assessed by asking the caregivers to indicate how many times they provided meals and snacks to the patients. A qualitative 24-hour dietary intake recall was used to determine the adequacy of the patient’s diets. A dietitian assessed all of the foods and drinks that a patient consumed before the nutrition consultation intervention (baseline) and after the intervention (follow-up).

Protein and energy intakes were calculated in grams and kilocalories, respectively, based on the NUFOOD system [13] and the Taiwan food composition table [14]. Data was retrospectively collected using a nutrition care list filled in by a dietitian and discussed with the caregivers. When a patient consumed anything in addition to the hospital menu this was documented precisely by the dietitian.

Other general and medical information, anthropometric data, and information on additional nutrition was obtained from either electronic or written hospital records by using a structured case record form.

### 2.5. Statistical analysis

The data obtained on food and nutrient intake was then analyzed statistically. Mean and standard error were calculated for each variable. Average daily energy and protein intake by the subjects before and after nutrition education were analyzed by repeated measures ANOVA. McNemar’s test was used to compare the difference in the proportion of energy or protein between the baseline and intervention. Statistical analysis was conducted using SPSS 16.0 (SPSS, Inc., Chicago, IL, USA). A value of *P* < 0.05 was considered to be statistically significant.

**Table 1:** - Patient characteristics and nutritional assessment results for all 444 patients. (Total number and percentage of patients)

High risk of undernourished (MUST ≧ 2)	Total (n)	Total (%)
**Gender**		
Female	109	24.55
Male	335	75.45
**Record malnutrition risk category BMI** **(kg/m** ^2^ **)**		
< 18.5 (score = 2)	257	57.88
18.5-20 (score = 1)	38	8.55
> 20 (> 30 obese) (score 0)	149	33.55
**Weight loss (unplanned wt loss in 3~6 mo)**		
> 10% (score = 2)	114	25.67
5-10% (score = 1)	133	29.95
< 5% (score 0)	197	44.36
**Acute disease effect**		
NPO > 5 days (score = 2)	45	10.13
**Main types of cancer**		
Squamous cell carcinoma	85	19.14
Oral/tongue cancer	58	13.06
Lung cancer	52	11.71
Intestinal cancer	36	8.11
Esophageal cancer	35	7.88
Liver cancer	23	5.18
Pancreas/gall cancer	23	5.18
Nasopharyngeal carcinoma NPC	22	4.95
Gastric cancer	18	4.05
Breast cancer	16	3.60
Cervix/ovarian cancer	15	3.38
Others	61	13.74

## 3. Results

### 3.1. Nutritional parameters

**Fig. 3:**
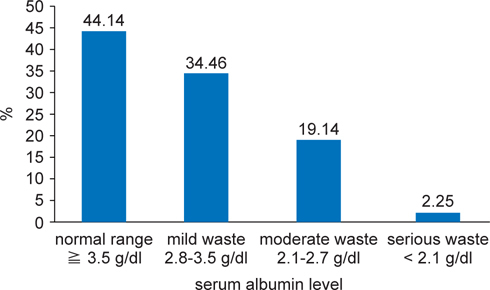
- Bar chart showing serum albumin in high risk cancer patient (n = 444).

All patients (n = 444) were screened using MUST, enabling malnutrition risk. All patients were at risk of malnutrition. 66.4% (n = 295) of patients had a BMI < 20 kg/m^2^ (BMI score ≧ 1). 55.6% (n = 247) had unintentional weight loss in 3-6 months > 5% (weight loss score ≧ 1). 10.1% (n = 45) had an ‘acute disease effect’ NPO > 5 days (score = 2). Table 1 shows the results of the nutritional parameters using MUST to screen. We also watched the serum albumin level: 44.14% (n = 196) of patients had a normal range (Alb ≧ 3.5 g/dl); 34.46% (n = 153) of patients had a mild waste (Alb 2.8~3.5 g/dl); 19.14% (n = 85) of patients had a moderate waste (Alb 2.1~2.7g /dl); and 2.25% (n = 10) of patients had a serious waste (Alb < 2.1 g/dl). Figure 3 shows the results of the nutritional parameters of serum albumin.

**Table 2:** - Average daily food intake by the subjects before and after nutrition education

	Mean	±SD	Mean	±SD	P-value^†^
Energy intake (Kcal/D)	1098.15	539.42	1578.90	454.74	0.000**
Protein intake (gm/kg)	0.76	0.41	1.16	0.40	0.000**

^†^Repeated measure ANOVA; adjustment gender, BMI, serum albumin.

**P < 0.01, significant at *P* < 0.05.

**Table 3:** - Different levels between before and after nutrition education of patient’s energy and protein

	**Before nutrition education**		**After nutrition education**		
**N = 444**	**N**	**%**	**N**	**%**	**P-value** ^†^
Energy intake (Kcal/D)					0.000**
< 500	62	13.96	12	2.703	
500-1000	107	24.10	42	9.459	
1001-1500	208	46.85	129	29.054	
> 1500	67	15.09	261	58.784	
Protein intake (gm/kg)					0.000**
< 0.6	135	30.41	30	6.76	
0.6-0.8	153	34.46	86	19.37	
0.9-1.2	105	23.65	121	27.25	
> 1.2	51	11.49	207	46.62w	

^†^McNemar’s test.

**P < 0.01.

### 3.2. Protein and Energy Intake

There were 527 cancer patients that MUST screened as high risk. Of those, 83 patients did not finish the follow-up and were excluded from this study. The average daily intake of energy and proteins were 1098.15 ± 539.42 Kcal/D and 0.76 ± 0.41 g/Kg BW respectively before imparting nutritional education. The increase in intake was found statistically significant after imparting the nutritional education, with daily intake of energy and proteins becoming 1578.90 ± 454.74 Kcal/D and 1.16 ± 0.40 g/Kg BW respectively (P < 0.0001) (Table 2). McNemar’s test shows a statistically significant difference between before and after nutrition education in patients’ energy and protein intake at different levels. Before nutrition education, the daily energy intake was < 500 Kcal 13.96% (n = 62), 500-1000 Kcal 24.1% (n = 107), 1001-1500 Kcal 46.85% (n = 208), and > 1500 Kcal 15.09% (n = 67). And after nutrition education, the daily energy intake improved to <500 Kcal 2.703% (n = 12), 500-1000 Kcal 9.459% (n = 42), 1001-1500 Kcal 29.05% (n = 129), and > 1500 Kcal 58.78% (n = 261) *(P* < 0.0001) (Table 3). Protein intake before nutrition education was < 0.6 gm/kg 30.41% (n = 135), 0.6-0.8 gm/kg 34.46% (n = 153), 0.9-1.2 gm/kg 23.65% (n = 105), and > 1.2 gm/kg 11.49% (n = 51). After nutrition education, daily protein intake improved to < 0.6 gm/kg 6.76% (n = 30), 0.6-0.8 gm/kg 19.37% (n = 86), 0.9-1.2 gm/kg 27.25% (n = 121), and > 1.2 gm/kg 46.62% (n = 207) (P < 0.0001) (Table 3).

## 4. Discussion

Malnutrition is common in cancer patients and has a negative impact on disease outcome. Malnutrition increases the duration of the hospital stay [15], reduces the cost-benefit and risk-benefit ratios of anticancer treatments [15], and is directly or indirectly responsible for excess mortality among cancer patients [16]. In an adult the normal range of serum albumin is defined as 3.55.0 g/dl and a level < 3.5 g/dl is called hypoalbuminemia [17]. Hypoalbuminemia has been demonstrated to more reliably reflect protein-energy malnutrition than anthropomorphic markers in many studies [18]. There is convincing evidence that the lower the serum albumin level, the higher the risk for postoperative complications and death [19]. Our data show that hypoalbuminemia of our patients at 55.85% (Figure 3).

The guidelines of the European Society for Clinical Nutrition and Metabolism (ESPEN) state that nutritional screening should be able to predict the clinical course based on nutritional status and whether a patient could benefit from nutritional treatment [7]. Screening tools are planned to detect protein and energy malnutrition and/or to predict whether malnutrition is likely to develop or deteriorate under present and future circumstances affecting a patient. In hospitals, further aspects of a disease have to be considered in combination with nutritional measurements in order to determine whether nutritional support is likely to be beneficial. The purpose of the MUST system is to detect adults who are at risk for malnutrition or who are malnourished on the basis of knowledge about the association between impaired nutritional status, body composition, and physical function (Figure 1) [9].

The aim of this study was to investigate whether nutrition education improved protein and energy intakes in undernourished hospitalized cancer patients (Figure 2). The goals of nutritional support in patients with cancer are numerous and include maintaining an acceptable weight and preventing or treating proteincalorie deficiencies, leading to better tolerance of treatment and its side effects, more rapid healing and recovery, reduced risk of infection during treatment, and enhanced overall survival [20]. A systematic review and meta-analysis of oral nutritional interventions in malnourished cancer patients by Baldwin *et al.* showed that nutritional intervention, including nutritional counseling and oral nutritional supplementation, was associated with statistically significant improvements in weight and energy intake compared with routine care (mean difference in weight = 1.86 kg, 95% CI = 0.25 to 3.47, *P* = 0.02; and mean difference in energy intake = 432 kcal/d, 95% CI = 172 to 693, *P* = 0.001) [21].

In this study oral nutritional interventions provided to highrisk cancer patients significantly improved their nutritional status and the quality of the diet consumed, and was associated with statistically significant improvements in protein and energy intake compared with the baseline (mean difference in protein = 0.4 g/d, *P* < 0.01; and mean difference in energy intake = 480 kcal/d, *P* < 0.01) (Table 2).

When a patient reported symptoms such as constipation, poor appetite, and abdominal pain, the dietitian advised the patient to consume frequent small meals, provided tips for treatment, and provided detailed explanations on food preparation skills to the caregiver in order to increase the nutrition density in food and to prepare a balanced liquid diet. Hutton *et al.* [22] reported lower energy intake (by 900-1,000 kcal/day), higher rates of weight loss, and lower patient’s quality of life (QOL) scores in patients with severe chemotherapy-associated chemosensory distortions. Cachectic patients should be supplemented with 1000-1500 calories per day (20-25 kcal/kg per day for bedridden patients and 25-30 kcal/kg per day for ambulatory patients) in the form of a balanced essential amino-acid mixture, given between meals [23]. The Recommended Dietary Allowance (RDA) of 0.8 grams (g) of protein per kilogram (kg) of body weight per day is the amount of protein that adequately maintains nitrogen balance in healthy individuals, including the elderly [24]. For optimal dietary supplementation in cachexia, protein source and meal composition also need to be considered, but in practice, the optimal nitrogen supply for cancer patients cannot be determined at present. Protein levels of between 1.2 g and 2.0 g per kg body weight are required to maintain nutritional status according to Johnson [25].

In this study, intervention aimed at a protein and energy intake of 1.2 g/kg and at least 1500 kcal/D respectively per day. Patient’s energy intake went from 1500 kcal/day for 15.09% (67 n) at baseline to 58.78% (261 n) after intervention *(p* < 0.01), and the protein intake went from 1.2 g/kg/day for 11.49% (51 n) at baseline to 46.62% (207 n) after intervention *(p* < 0.01) (Table 3).

There are limitations in the implementation of our nutrition education intervention as well as the evaluation instrument that may have influenced the study findings and generalization. Differences in exposure to nutrition information, family support, and food availability and accessibility could influence the patients’ responses to the nutrition education intervention. There may also have been limitations related to the nutrition knowledge, attitude, and food habits as an evaluation instrument. Moreover, this study was limited by the small sample size, with only inpatient participants, and the short duration of study time, which could be considered as being not generalized enough. We suggest further research on a larger sample size and more varieties of participants as well as developing more specific strategies and finding comparative nutrition changes among outpatients.

## Implications for research and practice

Nutrition intervention in cancer patients can involve many strategies, including dietary counseling and oral nutritional supplementation. Studies concerning the consumption of foods by hospital oncology patients are necessary to establish a relationship between intake values and organic levels, including the checking of the specific nutritional requirements, dealing not only with those on enteral and parenteral diets, but also those on oral hospital diets, who represent the great majority of hospital patients. It is strongly supported that nutritional education can be used as an effective measure to bring about favorable and significant changes in the dietary patterns of hospital oncology patients.

## Acknowledgments

This study was supported by a grant from the Chung Shan Medical University Hospital, (CSMUH IRB No: CS11124), Taiwan. We would like to express our sincere appreciation to the subjects for their participation and to all clinical registered dietitians (RD), who kindly provided the supplements for this trial. We also thank the nurses at the cancer care ward for providing expert assistance regarding the Nutritional Screening Project. All other authors declare no conflict of interest.

## Conflict of interest

None of the authors reports a conflict of interest.
